# Detecting early safety signals of infliximab using machine learning algorithms in the Korea adverse event reporting system

**DOI:** 10.1038/s41598-022-18522-z

**Published:** 2022-09-01

**Authors:** Jeong-Eun Lee, Ju Hwan Kim, Ji-Hwan Bae, Inmyung Song, Ju-Young Shin

**Affiliations:** 1 School of Pharmacy, Sungkyunkwan University, 2066, Seobu-ro, Jangan-gu, Suwon, Gyeonggi-do 16419 Republic of Korea; 2 Department of Health Administration, College of Nursing and Health, Kongju National University, Gongju, Republic of Korea; 3 Samsung Advanced Institute for Health Sciences & Technology, Sungkyunkwan University, Seoul, Republic of Korea; 4 Department of Biohealth Regulatory Science, Sungkyunkwan University, Seoul, Republic of Korea

**Keywords:** Epidemiology, Adverse effects, Data mining, Machine learning

## Abstract

There has been a growing attention on using machine learning (ML) in pharmacovigilance. This study aimed to investigate the utility of supervised ML algorithms on timely detection of safety signals in the Korea Adverse Event Reporting System (KAERS), using infliximab as a case drug, between 2009 and 2018. Input data set for ML training was constructed based on the drug label information and spontaneous reports in the KAERS. Gold standard dataset containing known AEs was randomly divided into the training and test sets. Two supervised ML algorithms (gradient boosting machine [GBM], random forest [RF]) were fitted with hyperparameters tuned on the training set by using a fivefold validation. Then, we stratified the KAERS data by calendar year to create 10 cumulative yearly datasets, in which ML algorithms were applied to detect five pre-specified AEs of infliximab identified during post-marketing surveillance. Four AEs were detected by both GBM and RF in the first year they appeared in the KAERS and earlier than they were updated in the drug label of infliximab. We further applied our models to data retrieved from the US Food and Drug Administration Adverse Event Reporting System repository and found that they outperformed existing disproportionality methods. Both GBM and RF demonstrated reliable performance in detecting early safety signals and showed promise for applying such approaches to pharmacovigilance.

## Introduction

Post-marketing surveillance studies are essential in ensuring drug safety through a periodic monitoring of the potential adverse events (AEs) that were not identified during clinical trials^1,2^. Routine drug safety monitoring has traditionally been based on a spontaneous reporting system (SRS) by applying statistical data mining tools to promptly identify a safety signal, that is an AE related to a specific drug that requires further investigation on a causal relationship^3,4^. This in turn enables the health authorities to take timely actions to mitigate further safety risks through a regulatory action^5,6^.

Many data mining methods are available to detect safety signals in SRS, including frequentist-based methods (i.e., proportional reporting ratio [PRR] and reporting odds ratio [ROR]) and Bayesian approaches (i.e., gamma Poison shrinkage model [GPS] and information component [IC] of Bayesian confidence propagation neural network [BCPNN])^7–9^. Performances of these methods have been explored previously, with the latter generally demonstrating higher sensitivity and specificity^10^. Recently, there has been a growing attention on the application of machine learning (ML) algorithms in pharmacovigilance^11^. Among studies that explored ML-based AE prediction, one study applied an ensemble ML algorithm trained with the AE profiles extracted from the US Food and Drug administration (FDA) Adverse Event Reporting System (FAERS) to identify 18 of 23 AEs identified during post-marketing surveillance^12^. Another study utilized information on the known AEs and drug indications extracted from the Side Effect Resource (SIDER) database to train ML algorithms, and successfully predicted AEs responsible for a drug withdrawal^13^. Besides AE prediction, few studies also have utilized ML algorithms to identify safety signals in the SRSs^14,15^. Preliminary evidence has shown that ML algorithms were not superior over frequentist-based methods and Bayesian approaches in detecting safety signals; however, this may be attributed to sparseness of certain combination of drug and AE leading to poor representativeness of ML training datasets, which may negatively impact the performance of ML algorithms^16^.

While the value of the data mining methods has been well established in the SRS databases, relatively little is known on the performance of ML algorithms in post-marketing surveillance. Indeed, ML algorithms had been utilized in predicting potential drug-AE association with the processing of natural languages from social media or health-related forums^17,18^; however, these approaches were laborious, requiring annotations of the lay languages by qualified medical experts^11^. Contrastingly, information in the SRS databases is based on standardized international medical terminology or classification systems, thereby requiring less human input for constructing training datasets.

The Korea Adverse Event Reporting System (KAERS) database was established in 1988 and managed by the Korea Institute of Drug Safety and Risk Management (KIDS)^19^. Similar to FAERS, it contains spontaneous reports of suspected drug and AE (s) reported by the healthcare professionals, consumers, and pharmaceutical companies. All AE reports are processed and stored as an individual case safety report that are periodically submitted to regulatory bodies (i.e., Ministry of Food and Drug Safety, World Health Organization (WHO) - Uppsala Monitoring Centre) or provided for research use. Many efforts have been made to detect safety signals in the KAERS, applying a variety of data mining methods including frequentist-based methods^20–22^, Bayesian approaches and tree-based scan statistics^23,24^.

Given the growing interest, this study aimed to investigate the utility of ML algorithms on early detection of AEs in the KAERS. Our recent pilot study successfully identified new safety signals for two anti-neoplastic drugs, nivolumab and docetaxel, using ML algorithms^15^. Here, we expand from our previous work to explore the supervised ML-based early AE prediction, using KAERS data and drug label information as the feature and label data for ML training, respectively. Two ensemble ML algorithms, gradient boosting machine (GBM) and random forest (RF), previously demonstrated to be the most effective in classifying safety signals using real-world data^14^, were applied to detect early safety signal, defined as an AE detected prior to it being updated in the drug label information. Specifically, we selected 5 AEs identified during post-marketing surveillance of a case drug, infliximab, an TNF-alpha inhibitor commonly prescribed for rheumatoid arthritis.

## Results

### Characteristics of the AE reports

Of 11,376 AE reports, 4482 included infliximab as the suspected drug. Gender was relatively evenly distributed (44.7%, 43.0% for men and women, respectively), and 30.9% of the reports were from age between 30 and 49 years. The report volume increased gradually throughout the study period, with a notable peak of 35.9% in 2016, and 28.0% of the reported AEs were categorized as serious AE. The majority were reported through post-marketing surveillance (60.6%), by manufacturer (83.3%), and from physician (74.8%), implying that these were filed from physician to manufacturer and subsequently got submitted as post-marketing surveillance reports to the KAERS (Table 1).

**Table 1:** Characteristics of the AE reports of infliximab and methotrexate in the KAERS between 2009 and 2018.

Characteristics	Infliximab		Methotrexate		*P* value
	N = 4482	100.00 (%)	N = 6894	100.00 (%)	
**Gender**					< .0001
Male	2002	44.7	2563	37.2	
Female	1929	43.0	4144	60.1	
Unknown	551	12.3	187	2.7	
**Age group (year)**					< .0001
< 20	413	9.2	1635	23.7	
20–29	486	10.8	387	5.6	
30–39	735	16.4	611	8.9	
40–49	650	14.5	841	12.2	
50–59	666	14.9	1208	17.5	
60–69	449	10.0	960	13.9	
≥ 70	233	5.2	544	7.9	
Unknown	850	19.0	708	10.3	
**Report year**					< .0001
2009	16	0.4	68	1.0	
2010	77	1.7	219	3.2	
2011	136	3.0	290	4.2	
2012	122	2.7	642	9.3	
2013	264	5.9	852	12.4	
2014	521	11.6	747	10.8	
2015	631	14.1	686	10.0	
2016	1608	35.9	971	14.1	
2017	670	15.0	1137	16.5	
2018	437	9.8	1282	18.6	
**Serious AE**					< .0001
Yes	1255	28.0	1257	18.2	
**Report type**					< .0001
Spontaneous	1244	27.8	5605	81.3	
Post-marketing surveillance	2718	60.6	38	0.6	
Literature	404	9.0	542	7.9	
Others	116	2.6	709	10.3	
**Report Source by person**					< .0001
Physician	3351	74.8	1696	24.6	
Pharmacist	67	1.5	987	14.3	
Nurse	493	11.0	3126	45.3	
Consumer	112	2.5	96	1.4	
Healthcare professional	6	0.1	110	1.6	
Others	80	1.8	438	6.4	
Unknown	373	8.3	441	6.4	
**Report Source by Affiliation**					< .0001
RPVC	632	14.1	6124	88.8	
Manufacturer	3733	83.3	676	9.8	
Medical institution	37	0.8	85	1.2	
Pharmacy	1	0	5	0.1	
Consumer	0	0	3	0	
Others	79	1.8	1	0	

*AE* adverse event, *KAERS* Korea Adverse Event Reporting System, *RPVC *regional pharmacovigilance center.

### Primary analysis: early safety signal detection

Characteristics of the 5 pre-specified AEs of infliximab (i.e., agranulocytosis, cervical cancer, cerebrovascular accidents, leukemia, and transient visual loss) are described in Table S1. All AEs were reported by manufacturers and recorded as a serious AE except for transient visual loss.

**Figure 1:**
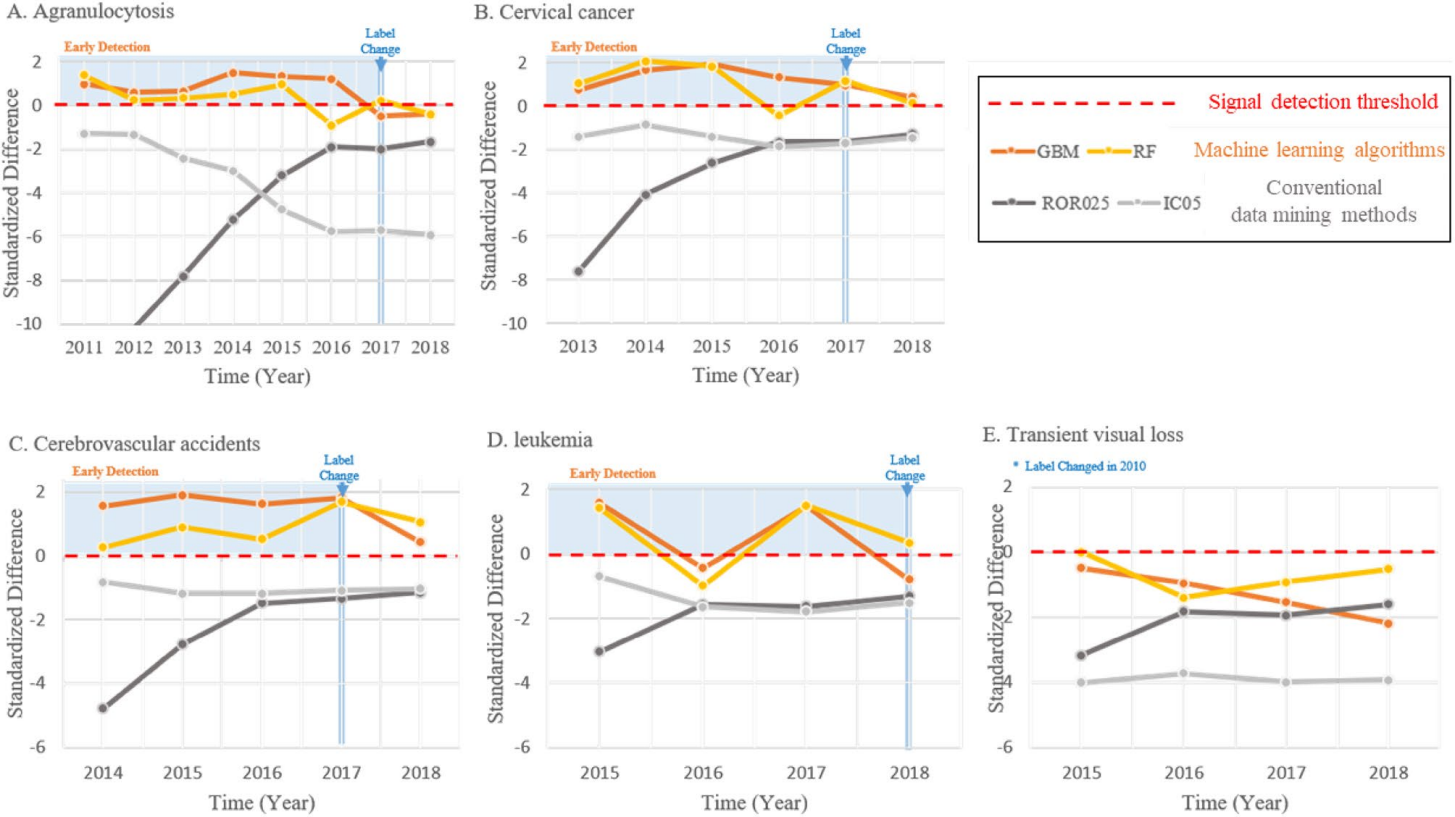
Standardized differences of data mining methods by calendar year for each pre-specified AE updated in the labeling information of infliximab Abbreviations: AE, adverse event; GBM, gradient boosting machine; RF, random forest; ROR025, adjusted reporting odds ratio; IC05, information component.

Of the 5 AEs assessed, RF and GBM identified 4 early signals, whereas adjusted ROR and IC did not identify any signal prior to the AEs being updated in the label information of infliximab (Table 2). The 4 early signals identified by RF and GBM were detected in the first year they were reported with infliximab in the KAERS (Fig. 1).

**Table 2:** Early signal detection results across different data mining methods in the KAERS between 2009 and 2018.

Drug	Adverse event term	WHO-ART preferred team	Label update (year)	Signaling prior to label update			
				RF	GBM	aROR	IC
Infliximab	Agranulocytosis	Agranulocytosis	2017	Y	Y	N	N
	cervical cancer	Cervical carcinoma	2017	Y	Y	N	N
	Cerebrovascular accidents	Cerebellar infarction	2017	Y	Y	N	N
		Cerebral infarction					
	Leukemia	Leukemia acute	2018	Y	Y	N	N
		Leukemia granulocytic					
	Transient visual loss	Vision abnormal	2010	N	N	N	N

*KAERS *Korea Adverse Event Reporting System, *WHO-ART* world health organization-adverse reaction terminology, *RF *random forest, *GBM *gradient boosting machine, *aROR *adjusted reporting odds ratio, *IC* information component.

- *Agranulocytosis*: WHO-Adverse Reaction Terminology (WHO-ART) Preferred Term “agranulocytosis” first appeared in the cumulative yearly dataset of 2009–2011. Both RF and GBM continuously identified this signal up to 2015, and the AE was updated in the label information of infliximab in 2017.
- *Cervical cancer*: WHO-ART Preferred Term “cervical carcinoma” first appeared in the cumulative yearly dataset of 2009–2013. Both RF and GBM continuously identified this signal up until it was updated in the label information of infliximab in 2017.
- *Cerebrovascular accidents*: WHO-ART Preferred Terms “cerebellar infarction” and “cerebral infarction” first appeared in the cumulative yearly dataset of 2009–2017. Both RF and GBM continuously identified this signal up until it was updated in the label information of infliximab, and the standardized difference values of GBM remained higher than that of RF throughout the early signal detection period.
- *Leukemia*: WHO-ART Preferred Terms “leukemia acute” and “leukemia granulocytic” first appeared in the cumulative yearly dataset of 2009–2015. Both RF and GBM identified this signal in the first year it appeared in the KAERS.
- *Transient visual loss*: WHO-ART Preferred Term “vision abnormal” first appeared in the cumulative yearly dataset of 2009–2015. Neither ML algorithms nor conventional data mining methods identified this AE throughout the data period.

### Performance of the data mining methods

**Figure 2:**
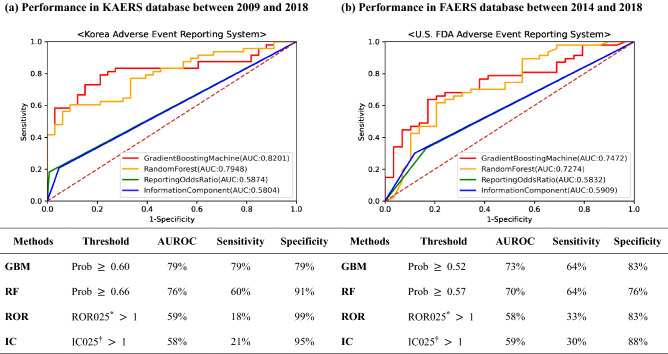
Receiver operating characteristic (ROC) curve illustrating the prediction performances of data mining methods used to detect safety signals of infliximab in KAERS (2009–2018) and FAERS (2014–2018). Abbreviation: KAERS, Korea adverse event reporting system; FAERS, FDA adverse event reporting system; AUROC, area under receiver operating characteristic curve; GBM, gradient boosting machine, RF, random forest; ROR, Reporting odds ratio; IC, Information component, Prob, probability. *ROR025 is the lower limit of a 95% confidence interval for estimated reporting odds ratio. †IC025 is the lower limit of a 95% confidence interval for estimated information component.

We measured the performance by comparing the signals detected by each data mining method with the reference standard. GBM demonstrated the best balance between sensitivity (79%) and specificity (79%), followed by RF with sensitivity and specificity of 60% and 91%, respectively. While the ROR and IC had higher specificities (ROR025: 99%; IC025: 95%), their sensitivities (ROR025: 18%; IC025: 21%) were considerably lower than that of ML algorithms. Performance measures expressed by area under receiver operating characteristics curves (AUROC) for the data mining methods are shown in Fig. 2.

### Secondary analysis: identifying new safety signals of infliximab

Among the unknown 148 AEs of infliximab in the KAERS, 27 safety signals were detected by both GBM and RF, whereas only 2 and 3 were detected by ROR025 and IC025, respectively (Table 3 and Table S2).

**Table 3:** Safety signal detection among the unknown AEs of infliximab reported in the KAERS between 2009 and 2018.

WHO-ART preferred term	Data mining methods							
	GBM		RF		aROR		IC	
	Signal	Probability	Signal	Probability	Signal	ROR025^a^	Signal	IC05^b^
Acne	Y	0.92	Y	0.74	N	0.64	N	− 0.33
Alopecia	Y	0.94	Y	0.77	N	0.08	N	− 2.66
Asthenia	Y	1	Y	0.95	N	0.36	N	− 1.34
Bilirubinaemia	Y	0.93	Y	0.71	N	0.02	N	− 4.37
Cytomegalovirus colitis	Y	0.87	Y	0.75	N	0.2	N	− 1.07
Death	Y	0.88	Y	0.76	N	0.01	N	− 2.89
Drug reaction paradoxical	Y	0.88	Y	0.8	N	< 0.01	N	− 0.17
Epistaxis	Y	0.93	Y	0.81	N	0.78	N	− 0.63
Extravasation	Y	0.86	Y	0.73	N	0.03	N	− 2.11
Gastroenteritis	Y	0.97	Y	0.76	N	< 0.01	N	− 0.07
Haematuria	Y	0.99	Y	0.82	N	0.04	N	− 1.83
Hepatocellular damage	Y	0.88	Y	0.67	N	0.06	N	− 3.27
Hypoaesthesia	Y	0.9	Y	0.7	N	0.14	N	− 1.39
Liver fatty	Y	0.97	Y	0.79	N	< 0.01	N	− 0.17
Melaena	Y	0.95	Y	0.89	Y	2.79	Y	0.62
Mouth dry	Y	0.78	Y	0.65	N	0.26	N	− 1.06
Oedema genital	Y	0.9	Y	0.63	N	< 0.01	N	− 1.48
Oedema periorbital	Y	0.88	Y	0.64	N	0.11	N	− 1.99
Paraesthesia	Y	0.82	Y	0.83	N	0.83	N	− 0.16
Psoriasis	Y	0.87	Y	0.71	N	< 0.01	Y	0.19
Pulmonary infiltration	Y	0.97	Y	0.82	N	0.32	N	− 0.3
Stomatitis ulcerative	Y	0.89	Y	0.78	N	0.08	N	− 1.82
Stridor	Y	0.82	Y	0.65	N	< 0.01	N	− 1.48
Stupor	Y	0.84	Y	0.64	N	0.14	N	− 3.17
Temperature changed sensation	Y	1	Y	0.96	Y	4.23	N	− 0.08
Tremor	Y	0.85	Y	0.71	N	0.2	N	− 0.8
Uveitis	Y	0.66	Y	0.7	N	< 0.01	Y	0.08

*KAERS *Korea Adverse Event Reporting System, *WHO-ART* world health organization−adverse reaction terminology, *RF* random forest, *GBM *gradient boosting machine, *aROR *adjusted reporting odds ratio, *IC* information component.

^a^Lower bound of the 95% confidence interval of adjusted ROR.

^b^Lower bound of the 90% confidence interval of IC.

### Sensitivity analysis: validating the generalizability of ML algorithms

We further applied our models to data retrieved from the FAERS from 3rd quarter of 2014 (Q3 2014) to 4th quarter of 2018 (Q4 2018) and consistently found superior performance of ML algorithms. GBM consistently showed the best performance with sensitivity of 64%, specificity of 83% and overall AUROC of 75% (Fig. 2). RF achieved the second highest overall AUROC (73%) with sensitivity of 64% and specificity of 76%, respectively (Fig. 2). Notwithstanding the relatively lower performance measures in FAERS than in KAERS, ML algorithms consistently performed better than ROR025 and IC025.

## Discussion

We applied supervised ML algorithms, GBM and RF, to explore their utility in early signal detection of post-marketing safety signals associated with infliximab in the SRSs. Among the 5 AEs with confirmed causality association to infliximab and added to the drug label information post-approval, GBM and RF identified 4 safety signals in the very first year these AEs appeared in the KAERS, whereas ROR and IC did not generate any early signals. According to the results of this study, ML algorithms performed better than the methods currently used by the regulatory agencies in South Korea (AUROC of 0.82 and 0.79 for GBM and RF, respectively, vs. 0.59 and 0.58 for ROR and IC, respectively). However, applying the same algorithms to FAERS data over a shorter time period of the infliximab lifecycle did not demonstrate the same performance, but they both outperformed ROR and IC respectively. The above findings show promise for applying such approaches to pharmacovigilance.

Our study successfully applied the ML algorithms in two SRSs, focusing on their ability to generate early signals. Both RF and GBM detected 4 out of 5 pre-specified AEs of infliximab as early as the very first year they were reported in the KAERS. However, our study also has some limitations. First, passive surveillance data are inherently subject to potential selection bias and underestimation of AE reports. Another potential bias may arise from duplication of the reports. Case duplicates, defined as “two or more reports describing the same occurrence of one or more AEs for the same patient that are assigned different case numbers instead of being linked as the same case”^25^, may potentially alter data mining calculations. However, our limited access to the SRS did not allow for deduplication of the data in hand. Second, potential misclassification of infliximab-AE pairs as either positive or negative control for reference standard construction in our study may had impact on the performance of the ML algorithms. Indeed, quantitative performance of ML algorithm is largely dependent on a quality of reference standard, and misclassification of the negative controls may certainly bias the performance measures in both directions^26^. However, we used the standard approach presented by the Observational Medical Outcomes Partnership (OMOP) researchers to reduce potential for such misclassification^27^. Third, the quantity and quality of an input data have major influences on performance of the ML algorithms and can lead to inconsistencies across different data sources^14^. In the sensitivity analysis for evaluating the generalizability of our findings, ML algorithms could not utilize all features available in the KAERS due to some differences in data characteristics between KAERS and FAERS; for example, information on the report type could not be identified in the FAERS database. Besides, information on an active ingredient of pharmaceutical product was not provided in the FAERS before Q3 2014, which led to the shorter data period for sensitivity analysis, compared with the main analysis. Therefore, the number of label positive and negative derived from FAERS was smaller than that derived from KAERS; Utilizing less samples and features might have resulted in achieving relatively lower performance of ML algorithms in the FAERS. Fourth, safety signals identified from the unknown AEs do not necessarily indicate causal relationship. These signals need to be prioritized by rarity or seriousness and require further investigation by the medical experts.

ML algorithm’s performance is largely determined by the quantity of the training dataset. Sparseness of certain combination of drug and AE due to relatively small report volume size in KAERS, compared with other large-scale SRS such as FAERS or VigiBase of WHO, may have limited the performance of the ML algorithms in our study. For instance, both GBM and RF failed to predict “transient visual loss” as early safety signal partly due to relatively low quantity of reports that included WHO-ART Preferred Term “vision abnormal”. Moreover, apart from low report count, none of the reports that listed “vision abnormal” were recorded as serious AE (Fig. S1). Given that serious AE designation in the KAERS was included in constructing covariate feature, it was likely that the ML algorithms yielded low signaling probability for this particular AE. Nonetheless, both RF and GBM successfully predicted 4 out of 5 AEs, promising as a new approach for early prediction of clinically significant AEs.

Expectedly, ML algorithms outperformed ROR and IC in predicting AEs as well as identifying new signals for infliximab. Such difference may be attributed to the statistical aspect of each method in calculating probability value for a drug-AE pair. Conventional data mining methods such as PRR, ROR, and IC simply calculate signal scores based on 2 × 2 contingency table^28^. While they are relatively simple and fast to compute, their performance varies across different thresholds and limited by high rate of false positives^29,30^. On the other hand, ML algorithms utilize a large number of features to calculate signal scores, and their performance is determined by the quality of input dataset. For instance, Schotland et al*.* used feature data generated by aggregating AEs from the FAERS and drug label information, sequentially, to construct an ensemble ML method, and tested whether unlabeled AEs at the time of drug approval could be predicted. The ML performance improved with increasing volume of input data, from precision, recall and specificity of 0.57, 0.78 and 0.61 with FAERS data to 0.67, 0.81 and 0.71 with addition of drug label information to the feature data, respectively^12^.

In the recent years, ML-based pharmacovigilance has been extended to studies that predict unlabeled AEs for a new drug at the time of approval by using the pharmacological target adverse event (TAE) profiles based on comparator drugs. An ensemble ML algorithm based on the data from drug label information, literatures and FAERS demonstrated reliable performance in predicting potential AEs of a new drug (AUC of 0.87)^31^. Besides ML-based AE prediction, they have been applied in routine safety surveillance for generating potential safety signals for evaluation^14,15^, as well as predicting unknown drug-drug interactions^32^. Our study findings complement the recently growing evidence on potential application of the ML algorithms, focusing on their ability to facilitate the timeliness of safety signal detection that would in turn reduce patient harm and improve health outcomes.

Of two ML algorithms, RF demonstrated optimal performance with better balance between sensitivity (74%) and specificity (89%), compared with 57% and 95% for GBM. Few studies have also assessed performances of these algorithms. One study based on Australian medication dispensing data showed GBM out-performed RF with sensitivities of 77% and 57%, respectively^14^. Another study compared performances of several data mining methods, including three ML algorithms, in which IC demonstrated the highest AUC of 0.69 and RF with the lowest AUC of 0.52^16^. Such discordance across different studies may attribute to the differential data volume, data characteristics, and lack of gold standard. Specifically, in context of differential data characteristics, there are currently no standardized guideline on mapping AEs listed in a drug label with the AE coding system used in each data source (i.e., WHO-ART used to code AE in the KAERS).

In conclusion, our study showed that the ML algorithms performed well in early detecting unknown AEs associated with infliximab. Before discussing the potential routine use of these methods in pharmacovigilance, additional efforts are needed to improve the consistency of the ML algorithms’ performance with other standardized drug-AE reference data. It is also vital to determine the acceptable performance levels in collaboration with the Korean and other regulatory agencies worldwide.

## Materials and methods

### Data source

Our study data source included AE reports in the KAERS between 2009 and 2018. Each report contains information on the demographic characteristics, administered drug (s), and suspected AE (s). Administered drug (s) are either labeled as “suspected drug”, “concomitant drug”, or “drug-drug interaction”. Each report may include one or more AEs, and classified as “serious AE” if they resulted in one or more of the following conditions: persistent or significant disability, congenital anomaly, life-threatening, death, hospitalization, prolonged hospitalized days, or other unspecified clinical intervention. Moreover, AEs reported by healthcare professionals or consumers are classified as “spontaneous”, pharmaceutical companies as “post-marketing surveillance” and monthly literature search for published case series or reports conducted monthly by KIDS as “literature”. AEs are coded according to the World Health Organization Adverse Reaction Terminology 092 (WHO-ART) and drugs according to the WHO’s Anatomical Therapeutic Chemical (ATC) classifications.

### Study scheme

**Figure 3:**
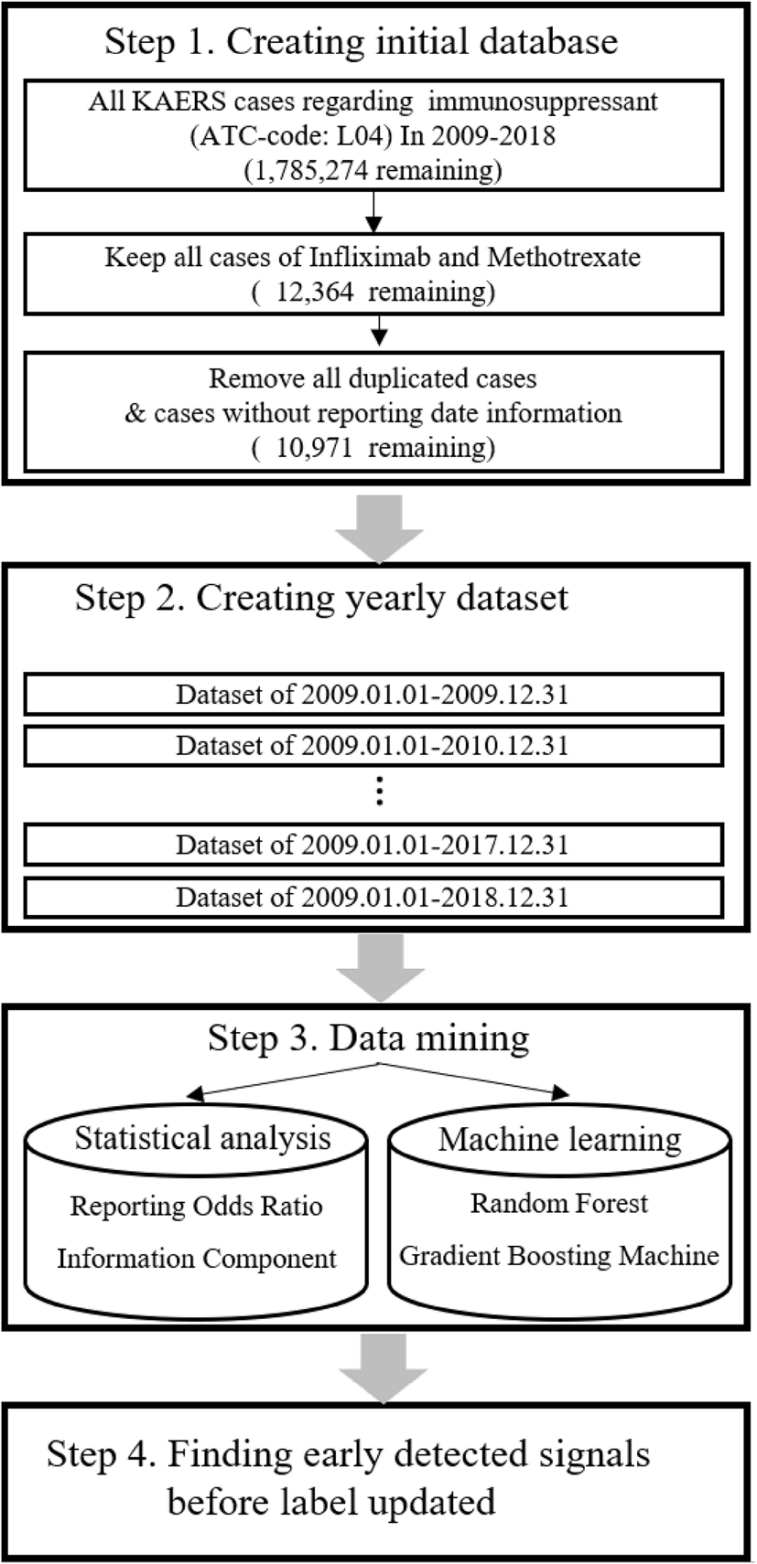
Step-by-step process from dataset construction to evaluation of the data mining methods in detecting early safety signals of infliximab in the KAERS between 2009 and 2018. Abbreviations: KAERS, Korea Adverse Event Reporting System; ATC, Anatomical Therapeutic Chemical Classification.

We used the ML algorithms and conventional data mining methods to detect safety signals for infliximab in the KAERS between 2009 and 2018. All AE reports that included two commonly prescribed disease-modifying antirheumatic drugs (i.e., infliximab and methotrexate) were analyzed to identify safety signals for infliximab. We first extracted all AE reports that contained immunosuppressants (ATC: L04) from the KAERS, and then created an initial dataset that listed infliximab or methotrexate as a “suspected drug”. For follow-up report of an initial report, only the latest reports were included. The initial dataset was segmented based on the reporting year, and 10 cumulative yearly datasets were created by merging each subsequent year data to the 2009 data (Fig. 3). Then, retrospective data screening was conducted from 2009 dataset to 2009–2018 dataset to evaluate whether the five pre-specified AEs of infliximab were detected prior to they were updated in the drug label information. After evaluating performance of each data mining method, we applied them to detect safety signals from unknown AEs in the KAERS that are neither listed in the drug label information of infliximab and other drugs belonging to same therapeutic class (i.e., TNF-alpha inhibitor).

### Generating input dataset for machine learning

We first constructed an input dataset required for ML algorithm to generate signaling probability of an AE. The input dataset contains information on the known and unknown AEs of a drug (labeled data) and quantifiable properties and characteristics of AEs (feature data).

#### Labeled data

Labeled data contains information on the known and unknown AEs of the study drugs. Known AEs, referred as reference standard, are retrieved from the drug label information and used to orient for training and testing of the ML algorithms. Unknown AEs are the ones that may be related the reported drug (s), for which causality relationship cannot be ruled out; this data is used by ML algorithms to detect new safety signals of the study drug.

To construct a labeled data, we first created a reference standard by extracting safety data of the study drug (i.e., infliximab) as well as other drugs belonging to the same therapeutic class (i.e., etanercept, adalimumab and golimumab) in the US FDA-approved drug label information; The labels (revised versions between 2009 and 2018) were obtained from the Labeling Archives of the National Library of Medicine DailyMed website^33^. Using the reference standard, AEs identified in the initial dataset were classified into the following categories: label-positive (Y), label-negative (N), and unknown AE (U). AEs labeled “Y” were the known AEs of the study drugs, “N” the ones not listed in both labeling information of the study drugs and other drugs belonging to same therapeutic class, and “U” the rest of the AEs in the initial dataset. The “Y” and “N” constituted the reference standard, and “U” the unknown AEs of the study drugs in the labeled data, respectively^14,34,35^. The numbers of AEs labeled “Y”, “N”, and “U” are presented in Table S2. Reference standard was used as an orientation for training and testing the ML algorithms.

#### Feature data

Supervised ML algorithms generate a signaling probability for each AE based on a feature data. We considered two aspects for selecting information to be included as the features: (1) minimizing potential bias due to missing data, and (2) enhancing the applicability of our methodology to other SRS databases. To take these into account, we used commonly required information for a valid individual case safety report (ICSR) described in “Guideline on good pharmacovigilance practice Module VI”: An identifiable patient, an identifiable reporter, a suspect drug, and an adverse event^36^. We generated a total of 35 features that included statistical, organ-specific, and covariate features. The statistical feature included the number of reports for a specific AE associated with infliximab from a 2 by 2 contingency table commonly used for disproportionality-based signal detection.

We also included the organ-specific features because typical AE is manifested or closely related to a specific organ, organ system, or mechanism of action. The system organ classes (SOCs) within the WHO-ART represents the category by which a specific AE is distinguished based on etiology, manifestation site, or purpose. A total of 32 SOCs were included as the organ-specific features, and 3 to 4 digits numeric codes (i.e., 100, 200…3200) of the SOCs were assigned a number from 1 to 32 to minimize the potential negative impact that the large numerical value might have on ML algorithms’ performance.

Covariate features included confounding factors, such as gender, age, report type, and report source by occupation and affiliation, which are commonly considered in the post-marketing safety surveillance studies^37,38^. Age was categorized into seven groups (0–20, 21–29, 30–39, 40–49, 50–59, 60–69, and older), consistent with a previous study on the safety signals of infliximab^39^. Variables for report type included spontaneous report, post-marketing surveillance study report, and case reports from literature. There were five (physician, pharmacist, nurse, other health professional, and consumer) and three (regional pharmacovigilance center, medical institution, and drug manufacturer) categories for report source by occupation and affiliation, respectively. Relative importance of features in signal detection using the ML algorithms are provided in Fig. S1.

### Primary analysis: early safety signal detection

We explored the performance of ML algorithms in detecting early safety signals through retrospective screening of the 10 cumulative yearly datasets. The algorithms were developed using each cumulative dataset to detect safety signals; for example, 2009–2012 data included AE cases reported between 2009 and 2012, with each AE classified as “Y”, “N” or “U” according to the reference standard constructed based on the drug labels updated in 2012. Then, AEs labeled “Y’ and ‘N’ in the 2009–2012 dataset were used to train and evaluate ML algorithms, and data mining methods were used to identify safety signals among the AEs labeled “U”.

Early signal was ascertained by whether it was detected prior to being updated in the drug label information. The five AEs selected a priori were the ones updated post-approval of infliximab through post-marketing surveillance after 2009 and described in details below:

- *Transient visual loss*: This term was listed on the infliximab label revised in 2010. WHO-ART Preferred Term “vision abnormal” was considered to be an equivalent term describing this AE.
- *Agranulocytosis*: This term was listed on the label revised in 2017. WHO-ART Preferred Term “agranulocytosis” was considered to be an equivalent term describing this AE.
- *Cervical cancer*: This term was listed on the label revised in 2017. WHO-ART Preferred Term “cervical carcinoma” was considered to be an equivalent term describing this AE.
- *Cerebrovascular accidents*: This term was listed on the label revised in 2017. WHO-ART Preferred Terms “cerebellar infarction” and “cerebral infarction” were considered to be an equivalent term describing this AE.
- *Leukemia*: This term was listed on the label revised in 2018. WHO-ART Preferred Terms “leukemia acute” and “leukemia granulocytic” were considered to be an equivalent term describing this AE.

#### ML algorithms for safety signal detection

Among the available ML algorithms, we chose two commonly used supervised classifiers, RF and GBM, which have been shown to demonstrate high specificity and positive predictive value in detecting AEs from large-scale medication dispensing data of Australia^14^.

- *RF*: The RF classifier is an ensemble model that combines the multiple decision tree classifiers of AEs. Many different subsets of a training dataset with randomly sampled features are used to train RF classifier. Therefore, it averages multiple deep decision trees which are trained on different parts of the same training dataset and eventually aims to reduce the variance and control over-fitting. RF had better predictive accuracy and performance in detecting signals than other supervised ML algorithms such as Support Vector Machine or Logistic Regression^14^.
- *GBM*: The GBM classifier is another ensemble classifier that combines many weak classifiers to generate stronger model. GBM begins by learning a base weak classifier and then generates the next model that reduces predictive errors of the precedent classifier. A stronger machine is eventually made as a result of the repeated process of developing the next model to minimize error.

Each algorithm produces an output value that ranges from 0 to 1, representing a probability of association between infliximab and an AE. When calculating signal detection performance using a reference standard, the signaling threshold is defined as a probability value that has the highest area under receiver operating characteristic curve (AUROC). AUROC is defined as:

$$AUROC = \frac{Sensitivity + Specificity}{2}.$$

#### Training ML algorithms

From the input data, we constructed gold standard dataset that contained label-positive (i.e., “Y”) and -negative AEs (i.e., “N”). Gold standard dataset was randomly divided into the training (75%) and test sets (25%), adjusting for imbalances in the distribution of the label-positive and -negative AEs with the Synthetic Minority Over-sampling Technique (SMOTE). We fitted RF and GBM with hyperparameters tuned on the training set by using a fivefold stratified cross-validation. Hyperparameter tuning is a strategy to optimize ML’s performance by identifying the optimal values of adjustable parameters in training process, such as the number of nodes in a decision tree to be made that affects the performance of decision tree. Five-fold stratified cross-validation is a resampling technique to evaluate ML algorithm on a finite sample by dividing data into five subsets with the same proportion of labels in the reference standard. The first fold was used as test set and the remaining as training sets, and repeated until all 5 folds were used as the test set.

#### Conventional data mining methods for safety signal detection

*Adjusted ROR*: Signal threshold of lower bound of 95% CI for adjusted ROR (ROR025) > 1 was used to detect safety signal^40^. We applied multivariate logistic model, expressed with the following formula, to present RORs adjusted for the potential confounder^41–45^:

$$Log \, \left( {odds} \right)\, = \,{\text{int}} ercept\, + \,\beta 1Y\, + \,\beta 2G\, + \,\beta 3A\, + \,\beta 4R_{t} \, + \,\beta 5 \, R_{s} \, + \,\beta 6 \, R_{a} \, + \,\beta 7 \, R_{s \, + } \beta 8 \, S$$

where Y = reporting year, G = gender, A = age-stratified group, R_t =_ report type, R_s_ = report source by occupation, R_a_ = report source by affiliation, S = serious AE.

*BCPNN*: The BCPNN is based on the Bayesian statistical principles for quantification of dependencies between drug and AE. Disproportionality that shows the dependencies calculated by BCPNN is called the Information Component (IC) and defined as:

$$IC = \log \cdot 2 \cdot \frac{Pxy}{{Px Py}},$$

where Px = probability that specific drug is a suspected drug in a case report, Py = probability that specific AE is reported in a case report, Pxy = probability that specific drug-AE combination is listed on a case report. IC > 0 indicates that a particular drug-AE combination is reported more frequently than expected in a dataset; the higher the value of the IC, the more relevant the specific drug-AE combination stands out in the database.

#### Comparing performance of the data mining methods

Given that signal detection thresholds differ across the data mining methods, we calculated standardized differences to compare performance between the methods. The standardized difference refers to a difference between predictive value and signaling threshold in the standard deviation unit, and it was calculated as:

$$d\, = \frac{Ps - To}{{SD}},$$

where Ps = Predictive value of an outcome of the signal detection algorithms, To = Optimal thresholds of the signal detection algorithms with the highest AUC, SD = Standard deviation which represents amount of variation or dispersion of the predictive values across all unknown AEs.

Infliximab-AE pairs with standardized difference > 0 were considered as the safety signals.

Also, we used the principles for evaluation of clinical diagnostic tests to quantify performances of the ML algorithms and conventional data mining methods in correctly differentiating AEs of infliximab. Reference standard was used to determine whether the safety signals were either true positives, false positives, true negatives, or false negatives to calculate sensitivity and specificity of each data mining method. Sensitivity was defined as the proportion of “label-positive” AEs that were correctly identified as signals (i.e., true positive/ [true positive + false negative]), and specificity as the proportion of “label-negative” that were correctly identified as non-signals (i.e., true negative/ [true negative + false positive]).

### Secondary analysis: identifying new safety signals of infliximab

Among the AEs designated “U” in the labeled data (unknown AE dataset), we used the ML algorithms to identify safety signals for infliximab. For each detected signal, we also calculated signal detection scores of ROR025 and IC05 to determine whether it was also detected by these data mining methods.

### Sensitivity analysis: validating the generalizability of ML algorithms

To validate our study findings, we further applied the ML algorithms to data retrieved from FAERS. First, we converted WHO-ART terms for defining AEs in the standard reference to Medical Dictionary for Regulatory Activities (MedDRA) terms. Then, we constructed an input dataset using AE reports in the FAERS from Q3 2014 to Q4 2018. Then, we applied ML algorithms to the input dataset to investigate drug-AE pairs in the standard reference and compared their predictive performance using the AUC of ROC plot.

All statistical analyses were performed using Python software version 3.7.5 (Python Software Foundation, Wilmington, DE, United States), SAS® software, version 9.4 (© 2002–2012 by SAS Institute Inc., Cary, NC, United States), and Microsoft Office 365 ProPlus (Microsoft Corp., Redmond, WA, United States). All methods used in this study were performed in accordance with the relevant guidelines and regulations.

### Ethical approval

The Institutional Review Board of Sungkyunkwan University (IRB no. 2019-04-020-001) approved this study and waived the need for an informed consent as only deidentified data were used.

## Supplementary Information

Supplementary Information.

## Data Availability

The proposed framework implemented using SAS and Python, along with results generated in the study are available in the “Early-detection” repository, https://github.com/SKKUPEPV/Early-detection.git. Our study used the Korea Adverse Event Reporting System (KAERS) database, established by the Korea Institute of Drug Safety & Risk Management (KIDS) in South Korea (Data number: 1905A0020). KIDS forbids the transfer, rent, or sale of the database to any third party other than the researcher, who obtained the approval for the provided database (Official website of KIDS: http://open.drugsafe.or.kr/; Contact information of data access committee: + 82-2-2172-6700). We accessed the data used in our study in the above mentioned manner, which we expect future researchers to do so in the same manner, and did not receive special privileges from KIDS.
