# Supplementary material for: Detecting early safety signals of infliximab using machine learning algorithms in the Korea adverse event reporting system
Source: Sci Rep. 2022 Sep 1;12:14869. doi: 10.1038/s41598-022-18522-z (PMC9436954; doi:10.1038/s41598-022-18522-z)
Supplement: Supplementary file 1 — Supplementary Information. [file 41598_2022_18522_MOESM1_ESM.docx]

**Detecting Early Safety Signals of Infliximab Using Machine Learning Algorithms in the Korea Adverse Event Reporting System**

Jeong-Eun Lee^1*^, Ju Hwan Kim^1*^, Ji-Hwan Bae^1^, Inmyung Song^2^, Ju-Young Shin^1,3^

^*^Contributed equally to this work as co-first authors

^1^School of Pharmacy, Sungkyunkwan University, 2066 Seobu-ro, Jangan-gu, Suwon-si,

Gyeonggi-do, South Korea

^2^ Department of Health Administration, College of Nursing and Health, Kongju National University, Gongju-si, South Korea

^3^Samsung Advanced Institute for Health Sciences & Technology, Sungkyunkwan University, Seoul, South Korea

**Corresponding Author**: Ju-Young Shin, PhD, Assistant Professor ([shin.jy@skku.edu](mailto:shin.jy@skku.edu))

School of Pharmacy, Sungkyunkwan University, 2066, Seobu-ro, Jangan-gu, Suwon, Gyeonggi-do 16419, Republic of Korea

**Supplementary Table 1.** Characteristics of the AEs of infliximab targeted for early signal detection in KAERS between 2009 and 2018

| **Characteristics** | **Agranulo-cytosis** | **Cervical cancer** | **Cerebro-vascular accident** | **Leukemia** | **Transient visual loss** |
| --- | --- | --- | --- | --- | --- |
| **Gender** |  |  |  |  |  |
| Male | 9 | 0 | 17 | 8 | 7 |
| Female | 0 | 16 | 7 | 10 | 0 |
| Unknown | 0 | 0 | 0 | 0 | 0 |
| **Age group (year)** |  |  |  |  |  |
| <20 | 0 | 0 | 3 | 0 | 0 |
| 20-29 | 0 | 0 | 0 | 0 | 0 |
| 30-39 | 9 | 0 | 0 | 8 | 0 |
| 40-49 | 0 | 0 | 0 | 0 | 4 |
| 50-59 | 0 | 0 | 14 | 4 | 0 |
| 60-69 | 0 | 0 | 0 | 0 | 0 |
| ≥70 | 0 | 0 | 7 | 0 | 3 |
| Unknown | 0 | 16 | 0 | 6 | 0 |
| **Report year** |  |  |  |  |  |
| 2009 | 1 | 0 | 0 | 0 | 0 |
| 2010 | 1 | 0 | 0 | 0 | 0 |
| 2011 | 0 | 0 | 0 | 0 | 0 |
| 2012 | 1 | 0 | 0 | 0 | 0 |
| 2013 | 1 | 1 | 0 | 0 | 0 |
| 2014 | 1 | 3 | 2 | 2 | 0 |
| 2015 | 1 | 3 | 4 | 4 | 1 |
| 2016 | 1 | 3 | 6 | 6 | 2 |
| 2017 | 1 | 3 | 6 | 5 | 2 |
| 2018 | 1 | 3 | 6 | 5 | 2 |
| **Serious AE** |  |  |  |  |  |
| Yes | 9 | 16 | 24 | 18 | 0 |
| **Report type** |  |  |  |  |  |
| Spontaneous | 0 | 0 | 3 | 0 | 0 |
| Post-marketing surveillance | 9 | 0 | 21 | 0 | 7 |
| Literature | 0 | 16 | 0 | 18 | 0 |
| Others | 0 | 0 | 0 | 0 | 0 |
| **Report Source by person** |  |  |  |  |  |
| Physician | 0 | 16 | 24 | 18 | 7 |
| Pharmacist | 0 | 0 | 0 | 0 | 0 |
| Nurse | 0 | 0 | 0 | 0 | 0 |
| Consumer | 0 | 0 | 0 | 0 | 0 |
| Healthcare professional | 0 | 0 | 0 | 0 | 0 |
| Others | 0 | 0 | 0 | 0 | 0 |
| Unknown | 9 | 0 | 0 | 0 | 0 |
| **Report Source by Affiliation** |  |  |  |  |  |
| RPVC | 0 | 0 | 0 | 0 | 0 |
| Manufacturer | 9 | 16 | 24 | 18 | 7 |
| Medical institution | 0 | 0 | 0 | 0 | 0 |
| Pharmacy | 0 | 0 | 0 | 0 | 0 |
| Consumer | 0 | 0 | 0 | 0 | 0 |
| Others | 0 | 0 | 0 | 0 | 0 |
| Abbreviations: AE, adverse event; KAERS, Korea Adverse Event Reporting System; RPVC, regional pharmacovigilance center. | | | | | |

**Supplementary Table 2.** Numbers of identified AEs included in the reference standard and unknown AEs in the KAERS between 2009 and 2018

| **No. of AEs identified in the KAERS** | **Reference standard** | | **No. of Unknown AEs^*^** |
| --- | --- | --- | --- |
|  | **No. of label-positive AEs ^†^** | **No. of label-negative AEs ^‡^** |  |
| 481 | 208 | 125 | 148 |
| Abbreviation: AE, adverse event; KAERS, Korea Adverse Event Reporting System.  ^†^AEs listed under WARNINGS AND PRECAUTIONS of the labeling information of infliximab or methotrexate.  ^‡^AEs not listed under WARNINGS AND PRECAUTIONS of the labeling information of infliximab, methotrexate, and other drugs in the same therapeutic class  ^*^AEs that are reported in the KAERS but not belonging to reference standard | | | |

**Supplementary Table 3.** Description of feature space for implementing machine learning algorithms

| **Features** | **Description** |
| --- | --- |
| **Statistical features** | |
| Feature a | The number of reports of a specific AE associated with the study drug |
| Feature b | The number of reports of other AEs related with the study drug |
| Feature c | The number of reports of a specific AE for comparative drugs |
| Feature d | The number of reports of other AEs for comparative drugs. |
| **Organ specific feature** | |
| System organ class | System organ class terms assigned to each adverse event in WHO-ART ver.092 |
| **Covariate features** | |
| Male | The number of reports that the patient’s gender is male among reports of a specific AE in the presence of the study drug |
| Female | The number of reports that the patient’s gender is female among reports of a specific AE in the presence of the study drug |
| Unknown_gender | The number of reports where the patient's gender cannot be identified among reports of a specific AE in the presence of the study drug |
| Age group under 20-year-old | The number of reports that the patient’s age is under 20 among reports of a specific AE in the presence of the study drug |
| Age group between 20 and 29 | The number of reports that the patient’s age is between 20 and 29 among reports of a specific AE in the presence of the study drug |
| Age group between 30 and 39 | The number of reports that the patient’s age is between 30 and 39 among reports of a specific AE in the presence of the study drug |
| Age group between 40 and 49 | The number of reports that the patient’s age is between 40 and 49 among reports of a specific AE in the presence of the study drug |
| Age group between 50 and 59 | The number of reports that the patient’s age is between 50 and 59 among reports of a specific AE in the presence of the study drug |
| Age group between 60 and 69 | The number of reports that the patient’s age is between 60 and 69 among reports of a specific AE in the presence of the study drug |
| Age group above 70-year-old | The number of reports where the patient's age group cannot be identified among reports of a specific AE in the presence of the study drug |
| Unknown age group | The number of reports where the patient's age cannot be identified among reports of a specific AE in the presence of the study drug |
| Non-SAE | The number of reports that the specific adverse event is not serious among reports of a specific AE in the presence of the study drug |
| SAE | The number of reports that the specific adverse event is serious among reports of a specific AE in the presence of the study drug |
| Spontaneous_reporting | The number of reports collected from spontaenous reports among reports of a specific AE in the presence of the study drug |
| Post_marketing_surveillance | The number of reports collected from post market surveillance studies among reports of a specific AE in the presence of the study drug |
| Literature | The number of reports collected by literatures among reports of a specific AE in the presence of the study drug |
| Unknown_report_type | The number of reports where the report type cannot be identified among reports of a specific AE in the presence of the study drug |
| Other_report_type | The number of reports where the type of reports cannot be identified among reports of a specific AE in the presence of the study drug |
| Physician | The number of reports reported by physicians among reports of a specific AE in the presence of the study drug |
| Pharmacist | The number of reports reported by pharmacists among reports of a specific AE in the presence of the study drug |
| Nurse | The number of reports reported by nurses among reports of a specific AE in the presence of the study drug |
| Other health professional | The number of reports reported by health professionals among reports of a specific AE in the presence of the study drug |
| Other_occupations | The number of reports reported by people from other occupations among reports of a specific AE in the presence of the study drug |
| Unknown_occupation | The number of reports where the reporter’s occuaption cannot be identified among reports of a specific AE in the presence of the study drug |
| Regional pharmacovigilance center | The number of reports reported by regional pharmacovigilance centers among reports of a specific AE in the presence of the study drug |
| Manufacturer | The number of reports reported by manufacturers among reports of a specific AE in the presence of the study drug |
| Medical_institution | The number of reports reported by medical_institutions among reports of a specific AE in the presence of the study drug |
| Pharmacy | The number of reports of a specific AE for the study drug from medical institutions |
| Other_affiliations | the number of reports reported by other affiliations among feature a. |
| Unknown_affiliation | the number of reports where the reporting source of affiliation cannot be identified among feature a. |


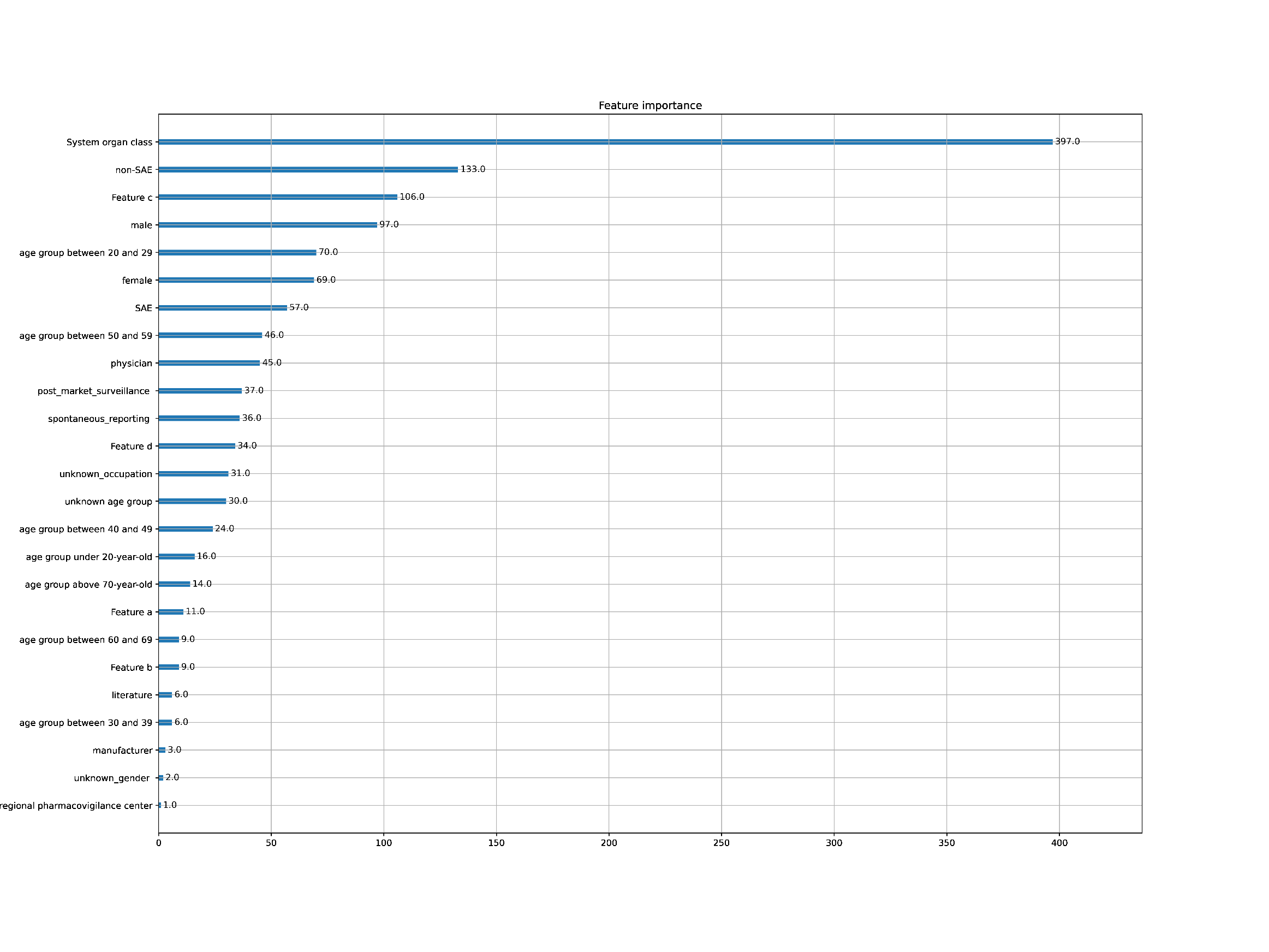


**Supplementary figure 1.** A plot for feature importance of statististical, organ-specific and covariate features in signal detection of infliximab using Gradient Boosting Machine

Abbreviations: SAE, serious adverse event
